# Synthesis, Theoretical Calculation, and Biological Studies of Mono- and Diphenyltin(IV) Complexes of *N*-Methyl-*N*-hydroxyethyldithiocarbamate

**DOI:** 10.3390/molecules27092947

**Published:** 2022-05-05

**Authors:** Jerry O. Adeyemi, Lukman O. Olasunkanmi, Adewale O. Fadaka, Nicole R. S. Sibuyi, Adebola O. Oyedeji, Damian C. Onwudiwe

**Affiliations:** 1 Department of Chemical and Physical Sciences, Faculty of Natural Sciences, Walter Sisulu University, Mthatha 5099, South Africa; aoyedeji@wsu.ac.za; 2 Department of Chemical Sciences, University of Johannesburg (Doornfontein Campus), Johannesburg 2028, South Africa; waleolasunkanmi@gmail.com; 3 Department of Chemistry, Faculty of Science, Obafemi Awolowo University, Ile-Ife 220005, Nigeria; 4 Department of Science and Innovation/Mintek Nanotechnology Innovation Centre, Biolabels Node, Department of Biotechnology, Faculty of Natural Sciences, University of the Western Cape, Private Bag X17, Bellville, Cape Town 7535, South Africa; afadaka@uwc.ac.za (A.O.F.); nsibuyi@uwc.ac.za (N.R.S.S.); 5 Material Science Innovation and Modelling (MaSIM) Research Focus Area, Faculty of Natural and Agricultural Science, North-West University (Mafikeng Campus), Private Bag X2046, Mmabatho 2735, South Africa

**Keywords:** dithiocarbamate, organotin complex, DFT calculation, cytotoxicity, anti-inflammatory assay

## Abstract

In this study, chlorophenyltin(IV) **[(C_6_H_5_)(Cl)Sn(L)_2_]** and diphenyltin(IV) **[(C6H5)2Sn(L)2]** of *N*-methyl-*N*-hydroxyethyldithiocarbamate were prepared and characterized using various spectroscopic methods (FTIR, ^1^H, ^13^C, and ^119^Sn NMR) and elemental analysis. The FTIR and NMR spectral data, used to establish the structure of the compounds, showed the formation of the complexes via coordination to the two sulfur atoms from the dithiocarbamate ligand and the respective phenyltin(IV) derivatives. This coordination mode was further explored by DFT calculations, which showed that the bonding around the Sn center in **[(C6H5)2Sn(L)2]** was more asymmetric compared to the bonding around **[(C_6_H_5_)(Cl)Sn(L)_2_]**. However, the Sn–S bonds in **[(C_6_H_5_)(Cl)Sn(L)_2_]** were found to be more covalent than those in **[(C6H5)2Sn(L)2]**. Furthermore, the charge density of the frontier orbitals showed that the Sn atom in the complexes is relatively electrophilic and the Sn atom in **[(C6H5)2Sn(L)2]** has a lower atomic dipole moment than that of **[(C_6_H_5_)(Cl)Sn(L)_2_]**. The cytotoxicity and anti-inflammatory study revealed that **[(C6H5)2Sn(L)2]**, with the higher number of phenyl substituents, has a higher potency than **[(C_6_H_5_)(Cl)Sn(L)_2_]**. The bio-efficacy study of these complexes as cytotoxic and anti-inflammatory agents showed that the complexes possessed moderate to high activity in comparison to the camptothecin and diclofenac in each case. Nevertheless, the diphenyltin(IV) derivative **[(C6H5)2Sn(L)2]** was found to possess a better activity than its counterpart due to the number of phenyl rings attached to the Sn center.

## 1. Introduction

Metal dithiocarbamate and its derivatives have continued to attract growing research attention due to their diverse structural variations, unique coordination chemistry, and wide biological properties [1,2,3,4,5,6]. Their organometallic derivatives such as the organotin(IV) dithiocarbamate complexes have been reported to possess interesting properties which include the possibility for ligand exchange, structural diversity, catalytic and redox capacity, and a variety of useful medicinal properties [7]. One characteristic feature of the organometallic compounds, which also makes them generally useful, is the presence of at least one metal–carbon bond within their organometallic moiety [8]. This has been found to contribute to the cytotoxic properties which are often associated with this class of compounds [8]. Organometallic compounds exhibit various structural motifs ranging from linear to octahedral, and even beyond. They have more stereochemical variations than most organic compounds (30 stereoisomers have been found for octahedral complexes with six different ligands), and the ability to control key kinetic properties by rational ligand design [8]. Furthermore, these compounds have been established to be uncharged, kinetically stable, and lipophilic in addition to the existence of their metal atoms in low oxidation states [8]. Thus, they offer the opportunity for the synthesis of novel complexes that possess specific modes of action outside the “classical coordination” in metal complex design. 

Organotin(IV) compounds have been widely used over the years for different biological purposes, but mostly as pesticides, fungicides, and anti-fouling agents in agriculture and various industries [9]. They have a special capacity for redox and catalytic reactions in addition to possessing a wide range of structural variations and a tendency for ligand exchange in biological systems [10]. Many derivatives of these compounds show potential as medicinal agents, although the mechanism of action in biology largely remains evasive. The often associated biological activity is thought to emanate from the number of Sn–C bonds, the nature of the alkyl/aryl derivatives, and the number of the alkyl/aryl derivatives present within the organotin moiety [11,12]. Organotin(IV) cation has been reported to be easily stabilized with ligands bearing different electronegative donor groups such as sulfur [12], which in turn confers useful biological properties upon complexation. Furthermore, the variation of the alkyl or aryl groups on the organotin backbone could impose notable biological activities [12]. Several organotin(IV) dithiocarbamate complexes have already been reported to show great potential as anticancer agents when examined using different human and cancerous cell lines [13,14,15]. In our group, we have studied the synthesis and the use of transition metal dithiocarbamate complexes for some biological applications [16,17]. Furthermore, in recent years, we have synthesized their organotin(IV) complexes and studied their cytotoxicity against many human cell lines, including human cervical carcinoma (HeLa) cells, human non-tumorigenic immortalized fibroblast (KMST-6), immortalized colorectal adenocarcinoma (Caco-2), and alveolar basal epithelial adenocarcinoma (A549) cells [18,19]. To this end, we recently studied a new class of dithiocarbamate bearing a hydroxyl group using a variation of alkyltin substituents [19]. Although many metal complexes have been reported for this dithiocarbamate ligand [20,21], their organotin(IV) counterparts have not been widely reported to the best of our knowledge. 

Therefore, in continuation of our previous work on hydroxyl bearing dithiocarbamate group, we examined the synthesis and characterization of mono- and diphenyltin(IV) derivatives of dithiocarbamate complexes and their biological potential (in vitro) as antioxidant and cytotoxic agents. To provide insights into the molecular and electronic structures of the complexes, and account for the observed properties, density functional theory (DFT) calculations were carried out for these complexes. 

## 2. Results and Discussion 

### 2.1. Synthesis and Characterization 

**Scheme 1:**
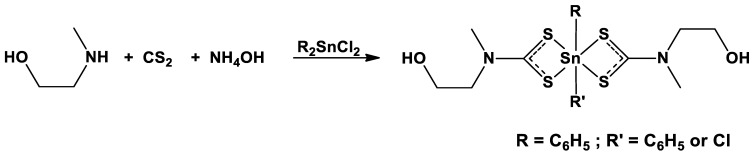
Synthetic scheme of the phenyltin(IV) *N*-methyl-*N*-hydroxyethyldithiocarbamate complexes.

The preparation of both complexes proceeded by means of a substitution reaction of the labile chloride ion of phenyltin(IV) chloride salts, as presented in Scheme 1. The percentage yields of the synthesized complexes **[(C6H5)2Sn(L)2]** and **[(C_6_H_5_)(Cl)Sn(L)_2_]** were 70% and 63%, respectively. The obtained complexes appeared slightly yellow in color, crystalline, stable in air, slightly soluble in methanol, dichloromethane, ethanol, and chloroform. 

The FTIR spectroscopy of the complexes showed three major regions often associated with the dithiocarbamate compounds: the stretching band frequencies attributed to *ν*(C=N), *ν*(C−S), and *ν*(M−S) in the regions 1580–1450, 1060–940, and 455–440 cm^−1^, respectively, as already established in the literature [22]. In the spectra of these complexes, the *v*(C=N) stretching vibration bands were found in the frequency range 1453–1456 cm^−1^ [23], which is between the stretching vibration of *ν*(C=N) and *ν*(C–N), that is 1690–1640 and 1350–1250 cm^-l^, hence suggestive of a partial double bond character [24]. Furthermore, the stretching *ν*(C−S) in this present study was found in both complexes as a single band between 990 and 988 cm^−1^, indicative of bidentate coordination between the metal and the dithiocarbamate ligand [23]. Additionally, the stretching bands attributed to *ν*(M−S), supporting the existence of coordination between the metal center and the ligand moiety, were found between 450 and 440 cm^−1^, and this confirmed the presence of Sn–S bonds in the complexes [25]

The proton (^1^H) NMR spectra of the different metal complexes and their derivatives with the ligand have already been reported in the literature [26], and the spectra obtained are presented in the Supplementary Data. The influence of the phenyl group on the metal center could be observed in the three main regions of the chemical shifts. Multiple signals in both complexes were found between 7.50 and 7.25 ppm, attributed to the phenyl groups of the organotin(IV) moiety, similarly to other previous reports [13]. Other notable protons, which conform with previous reports, the methylene proton signals found between 4.06 and 3.45 ppm, due to the deshielding effect of the attached electronegative O and N atoms [27,28]. Furthermore, the proton signals of the hydroxyl group for both complexes were found in the region between 7.90 and 7.80 ppm [19].

Similarly, the carbon signals associated with the carbon atom of the thioureide π-system δ(N^13^CS_2_) in each of the complexes were found around 200 pm in the ^13^C spectra (see Supplementary Data). The existence of this system has been found to contribute to the stability of complexes and which, in turn, results in the reduction in the electron density [29]. Other carbon atom signals found within the range of 150 and 124 ppm are associated with the carbons of the phenyltin(IV) derivatives similar to other reports [26,30]. The other three observed carbon atom signals found around 60, 59, and 45 ppm have all been attributed to the methylene and methyl carbons, respectively. The Sn signals were found at −467 ppm from the ^119^Sn NMR spectra obtained, which is indicative of a hexacoordinated geometry around the tin center [31].

### 2.2. Molecular and Electronic Structures

**Figure 1:**
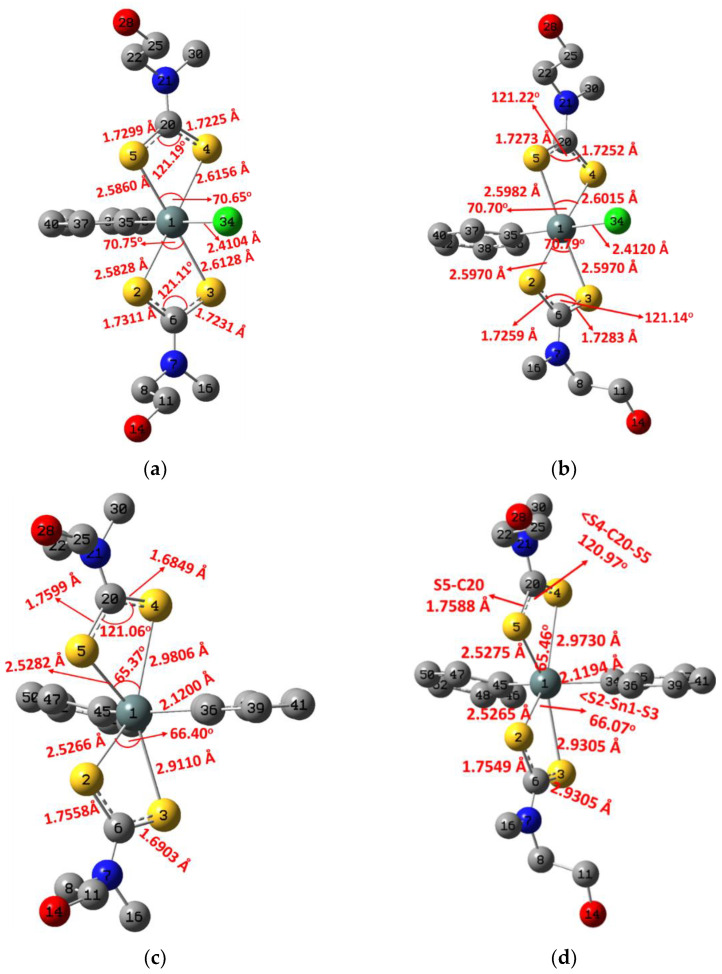
Optimized ground state equilibrium structures of (**a**) syn-syn conformation of **[(C_6_H_5_)(Cl)Sn(L)_2_],** (**b**) anti-anti conformation of **[(C_6_H_5_)(Cl)Sn(L)_2_],** (**c**) syn-syn conformation of **[(C6H5)2Sn(L)2]**, and (**d**) anti-anti conformation of **[(C6H5)2Sn(L)2],** obtained at M06/cc-TVPZ//LANL2DZ level.

The ground state equilibrium molecular structures of the studied Sn complexes are shown in Figure 1, which presents some salient bond lengths and angles. The possibility of rotation of ethanoyl and methyl groups on the dithiocarbamate ligands was considered such that two different conformations (**syn-syn or A and anti-anti or B**) are presented for each of the complexes. The conformer with the ethanoyl group on each ligand pointing in the same direction was found to have relatively lower energy than its counterpart. Accordingly, conformer **A** of **[(C_6_H_5_)(Cl)Sn(L)_2_]** is about 0.06 kJ/mol more stable than **B**, while conformer **A** of **[(C6H5)2Sn(L)2]** is about 0.56 kJ/mol more stable than **B**. The geometry parameters listed in Figure 1 fall within the range of experimental bond lengths and angles for tin dithiocarbamate complexes reported in the literature [32,33]. Similar to what was observed in the experimental IR data (vide supra Section 2.1), the predicted stretching vibration bands of the C–N bonds in **[(C_6_H_5_)(Cl)Sn(L)_2_]** range between 1410 cm^−1^ and 1513 cm^−1^ (unscaled), while similar bonds in **[(C_6_H_5_)(Cl)Sn(L)_2_]** showed predicted stretching vibration bands between 1404 cm^−1^ and 1507 cm^−1^. Furthermore, the C–N bond lengths for the two complexes are in the ranges of 1.3346(2) Å–1.3359(9) Å and 1.3385(3) Å–1.34118(1) Å, respectively, which are in-between the values for C–N single (ca. 1.482 Å, e.g., as in azetidine) and double (ca. 1.280 Å, e.g., as in diazirine) bonds [34]. These observations suggest that the C–N bonds in the complexes exhibit partial double bonds, which also correlates with the experimentally observed and inferred nature of the C–N bonds based on the experimental IR data. The four Sn–S bonds in **[(C_6_H_5_)(Cl)Sn(L)_2_]** are approximately 2.56 Å, indicating that the Sn center is covalently bonded to the S atoms of the dithiocarbamate chelates. However, the bond lengths for the four Sn–S bonds in **[(C6H5)2Sn(L)2]** range from 2.53 to 2.97 Å, exhibiting both covalent and coordinate bonding features in pairs. The observed features of the Sn–S bonds in **[(C6H5)2Sn(L)2]** are characteristic of tin dithiocarbamates with monodentate co-ligands [19,33]. The presence of organic groups as co-ligands (the phenyl groups in the case of **[(C6H5)2Sn(L)2]**) makes the dithiocarbamate ligand behave as a bidentate unsymmetric or anisobidentate ligand [33]. Unlike in the case of alkyl co-ligands, in which the geometry parameters of Sn-dithiocarbamates are not largely affected by the alkyl chain length [19], a comparison of the bond lengths and angles in **[(C_6_H_5_)(Cl)Sn(L)_2_]** and **[(C6H5)2Sn(L)2]** suggests that the nature of co-ligands (Cl and Ph) significantly affected the geometry parameters of the Sn-dithiocarbamate complexes. The Sn–S bonds in **[(C_6_H_5_)(Cl)Sn(L)_2_]** are relatively stronger than those in **[(C6H5)2Sn(L)2]** as the former is more covalent and showed a lesser degree of asymmetry in Sn-S bonds. More so, the C–S bonds in **[(C_6_H_5_)(Cl)Sn(L)_2_]** are shorter (stronger) than those in **[(C6H5)2Sn(L)2]**. The S–Sn–S angles in **[(C_6_H_5_)(Cl)Sn(L)_2_]** are larger than those in **[(C6H5)2Sn(L)2]**, while the S–C–S in both complexes is not significantly different. The shorter S–Sn–S bond angle in **[(C6H5)2Sn(L)2]** could be attributed to the bulkiness of the two phenyl co-ligands, which confers some strain on the structure. The dihedral angles between the planes of the pair of dithiocarbamate ligands through the Sn center are about 180° in both complexes, indicating co-planarity. 

The values of selected second-order perturbation/interaction energies (E^(2)^) between atomic orbitals resulting from natural bond orbital (NBO) calculations are listed in Table 1. The values of E^(2)^ recorded for ligand–Sn interactions revealed a strong overlap between the delocalized electronic orbitals of the S–C bonds of the dithiocarbamate ligands and the central Sn atom. An obvious trend in the values of E^(2)^ listed in the table is the higher values for the corresponding donor–acceptor interactions in **[(C6H5)2Sn(L)2]** compared to **[(C6H5)2Sn(L)2]**. This further confirms a stronger ligand–metal bond in the former than in the latter. Furthermore, the strong overlap was also recorded for S/Sn interactions based on lone pair donation from S orbitals to the unoccupied orbitals of Sn, as exemplified by the E^(2)^ values of the LP(3)S2 → LP*(2)Sn1 donor–acceptor interaction. Many of these LP/LP* interactions were observed in the complexes, though it was tricky to use them to explain the comparative bond strengths of the two complexes.

**Figure 2:**
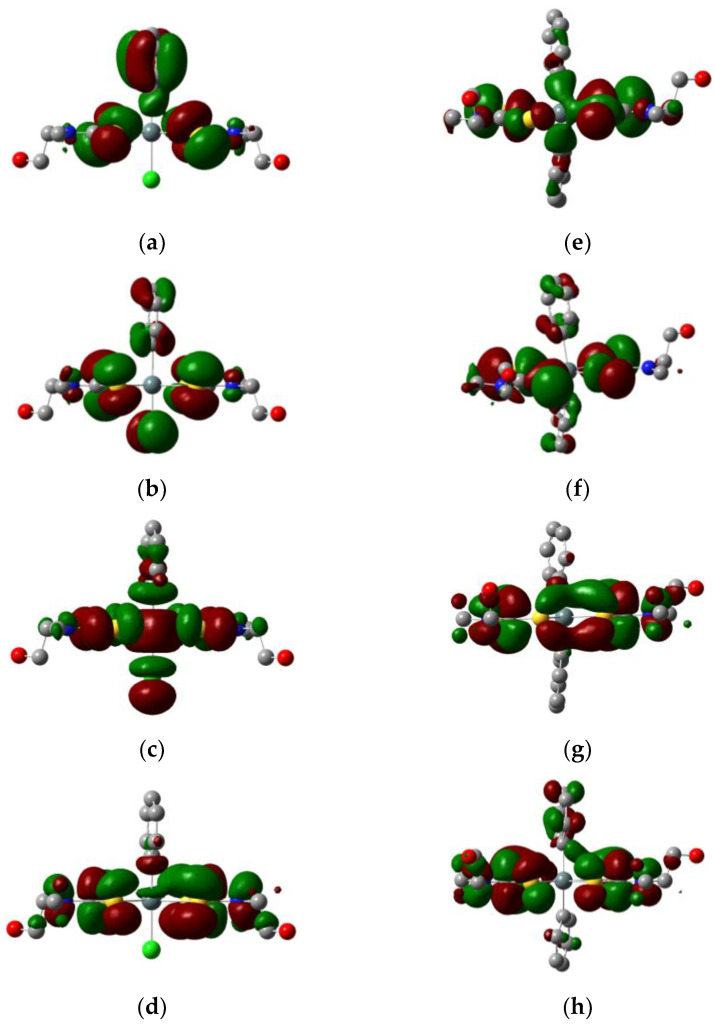
FMO isosurfaces of the complexes of **[(C_6_H_5_)(Cl)Sn(L)_2_]** (**a**) HOMO−1, (**b**) HOMO, (**c**) LUMO, (**d**) LUMO+1, and **[(C6H5)2Sn(L)2]** (**e**) HOMO−1, (**f**) HOMO, (**g**) LUMO, and (**h**) LUMO+1 (Iso-value = 0.020000).

Orbital composition analyses of the frontier molecular orbitals (FMOs) of both complexes were carried out with the aid of the Multiwfn software module [35,36] to print the percentage compositions of atomic orbitals involved in the FMOs. The iso-surfaces of the highest occupied molecular orbital (HOMO), lowest unoccupied molecular orbital (LUMO) as well as the HOMO-1 and LUMO+1 for both complexes are shown in Figure 2. It could be observed that the HOMO of **[(C_6_H_5_)(Cl)Sn(L)_2_]** is localized on the dithiocarbamate S-atoms, Cl atom, and the phenyl ring. The HOMO of **[(C6H5)2Sn(L)2]** is localized on the dithiocarbamate ligands and extended to the phenyl ring. The HOMO−1 canonical surfaces reflect similar distributions as the HOMO, but with more delocalization to other atoms. The Sn center does not make significant contributions to the HOMO and HOMO-1 orbitals in both complexes. The LUMO iso-surface of **[(C_6_H_5_)(Cl)Sn(L)_2_]** is delocalized over the Sn center, the dithiocarbamate, Cl, and a segment of the phenyl ligand, while that of **[(C6H5)2Sn(L)2]** is delocalized over the Sn center and dithiocarbamate ligands. The LUMO+1 iso-surfaces for both complexes exhibit lesser delocalization to the Sn center.

Numerical compositions (%) of the atomic orbitals for the FMOs are listed in Table 2. The HOMO of **[(C_6_H_5_)(Cl)Sn(L)_2_]** is mainly composed of ca. 98% p-orbitals of S atoms of dithiocarbamate ligands and Cl ligands. The compositions of S atoms to the HOMO range from 11 to 27%, while Cl atom contributes about 17%. Similarly, the HOMO-1 of **[(C_6_H_5_)(Cl)Sn(L)_2_]** is composed of ca. 97% p-orbitals of S atoms of dithiocarbamate ligands, as the contributions of S atoms range between 9% and 24%. The Sn center does not make any significant contribution to the HOMO and HOMO−1 of **[(C_6_H_5_)(Cl)Sn(L)_2_]**. The LUMO of **[(C_6_H_5_)(Cl)Sn(L)_2_]**, on the other hand, is composed of s-orbitals of Sn (ca. 39%) as well as the p-orbitals of Sn, S, and Cl atoms. The LUMO+1, however, has a lesser contribution from the Sn center (ca. 4%). The atomic compositions of FMOs in **[(C6H5)2Sn(L)2]** revealed asymmetric charge distribution, as the percentage contributions by similar pairs of atoms differ significantly. The S-atoms of the dithiocarbamate ligands contribute 11–44% to the HOMO, and 5–50% to the LUMO, both being mainly the p-orbitals of the atoms involved. The d-orbitals of S atoms make up about 8% of the total contributions by S atoms to the LUMO of the complex. The Sn center contributes ca. 3% to the LUMO of **[(C6H5)2Sn(L)2]**, while it makes only ca. 1.5% contributions to LUMO+1. The Cl co-ligand on the Sn center of **[(C_6_H_5_)(Cl)Sn(L)_2_]** is highly electron-withdrawing and renders the center electron-deficient, resulting in the high contribution of the Sn atom to the LUMO. However, the delocalized aromatic pi-electrons on the two C_6_H_5_ avails the Sn center in **[(C6H5)2Sn(L)2]** with some electron density, resulting in a low contribution to the LUMO. 

The relative lipophilic character of the complexes was adjudged by the atomic dipole moment of the central Sn atom. This character could be correlated with the biological activity of the complexes. A lower atomic dipole moment at the central metal suggests higher lipophilicity and a greater tendency to permeate through the cell wall. Atomic dipole moments of the Sn atoms in **[(C_6_H_5_)(Cl)Sn(L)_2_]** and **[(C6H5)2Sn(L)2]** were predicted to be 0.5859 a.u. and 0.4847 a.u., respectively, which suggests that **[(C6H5)2Sn(L)2]** with more phenyl substituents (lipophilic group) would be more biologically active than **[(C_6_H_5_)(Cl)Sn(L)_2_]**.

### 2.3. Biological Study

#### 2.3.1. Cytotoxicity Study

**Figure 3:**
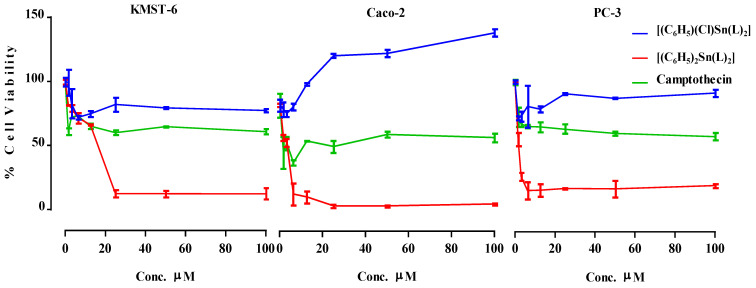
Cytotoxicity of the phenyltin(IV) complexes: **[(C6H5)2Sn(L)2]** and **[(C_6_H_5_)(Cl)Sn(L)_2_],** on cancer and non-cancer cells. Cell viability (%) was assessed by means of MTT assay after 24 h.

**Figure 4:**
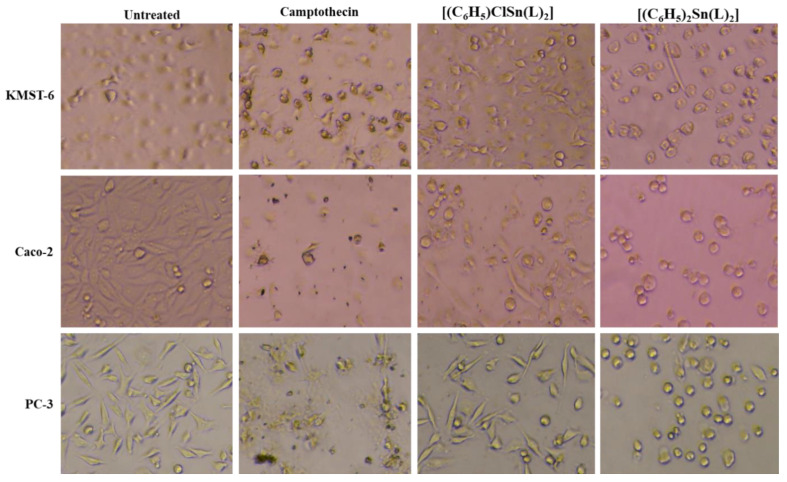
Effect of the phenyltin(IV) complexes **[(C6H5)2Sn(L)2]** and **[(C_6_H_5_)(Cl)Sn(L)_2_**] and Camptothecin on cellular morphology after 24 h treatment with 100 µM.

The cytotoxicity assay is generally considered a useful tool for the initial determination of the toxicity of test samples [37]. The cytotoxicity of the phenyltin(IV) complexes was studied against human immortalized cancer (Caco-2 and PC-3) and non-cancer (KMST-6) cells using MTT assay. Under the same conditions, the activity of the complexes was compared to that of camptothecin, which is a standard anticancer drug. The concentration of the complexes that inhibited 50% cell growth (IC_50_) was estimated after an incubation period of 24 h, and the IC_50_ of the complexes are summarized in Table 3. The activity of the complexes at various concentrations and the effect of the test complexes on the selected cell lines are presented in Figure 3 and Figure 4, respectively (using some representative images). The complex **[(C6H5)2Sn(L)2]** showed a superior activity and a largely better activity than the **[(C6H5)2Sn(L)2]**. This activity, in comparison to the standard drug (camptothecin) in all of the tested cell lines, showed that the diphenytin(IV) *N*-methyl-*N*-hydroxyethyldithiocarbamate is 5.5, >40, and 15.3 times more active in KMST, Caco-2; and PC-3 cell lines, respectively (Figure 3). This observed very good activity is in agreement with the previous report on the cytotoxicity study of organotin(IV)-based compounds [33]. The current study supports previous reports which suggest that, as the alkyl chain or aryl groups increases in an organotin compound, increased biological activity is often observed [38]. This has been attributed to Tweedy’s chelation theory, which affirms that complexation reduces the polarity of a metal ion, and in turn enhances the lipophilic properties of the complex, which consequently allows for the easy permeation of the complex through the cellular membranes [39]. Hence, as the aryl substituent increases from one in **[(C6H5)2Sn(L)2]** to two in **[(C6H5)2Sn(L)2]**, the polarity becomes lower, thereby resulting in increased lipophilicity of the complex. The increase in lipophilicity of the complex increases their cytotoxicity. Although the diphenyltin complex showed better activity than the mono-phenyl derivative and the standard drug, its nonspecific action toward both normal and cancer cell lines may be improved using drug carriers. This complex could be a potential lead anticancer drug, further studies and clinical screenings are warranted to investigate its anticancer mechanism and improvise strategies to increase the selectivity of the complex. 

#### 2.3.2. In Vitro Anti-Inflammatory Assay

Different studies have reported that most compounds that show cytotoxic properties possess some measure of anti-inflammatory activities [40,41,42]. Thus, the anti-inflammatory properties of the complexes were studied, and the results were compared to that of diclofenac as a standard drug. The IC_50_ values showed that the complexes **[(C6H5)2Sn(L)2]** (2.52 ± 0.02 µM) and **[(C_6_H_5_)(Cl)Sn(L)_2_]** (2.67 ± 0.03 µM) possess good anti-inflammatory properties, that is comparable and slightly better than the standard drug used (2.94 ± 0.01 µM). However, the diphenyltin(IV) derivative showed better activity compared to the mono-phenyltin(IV) derivative, which is in line with the generally observed trends from organotin(IV) compounds bearing aryl groups, due to increased lipophilicity emanating from reduced polarity around the tin metal center. Furthermore, the good activity found for these complexes has been attributed to the transportation of [(C_6_H_5_)_2_Sn(IV)]^+^ moiety through the cell membrane, due to the increased lipophilicity observed [43,44].

## 3. Materials and Methods

The alcoholic amine and all other reagents used in this study were acquired from Merck chemical Co. (Darmstadt, Germany), while the organotin(IV) salts were purchased from Sigma-Aldrich (Darmstadt, Germany). All of the reagents were used without further purification. Gallenkamp melting point apparatus was used to determine the melting point of the respective complexes. The percentage elemental composition (C, H, N, and S) was determined using Elementar, Vario EL Cube (Langenselbold, Germany). Furthermore, the proton, carbon, and tin (^1^H, ^13^C, ^119^Sn) spectra of the complexes were obtained from 600 MHz on a Bruker Avance III NMR spectrometer (Billerica, MA, USA), using tetramethylsilane as the internal standard at 25 °C. Additionally, an Alpha Bruker FTIR spectrophotometer (Billerica, MA, USA) was used to measure the infrared properties of the complexes. 

### 3.1. Synthesis of Organotin(IV) N-Methyl-N-hydroxyethyldithiocarbamate

An equal amount of a mixture of *N*–methyl–*N*–ethanolamine (0.8 mL, 0.01 mol), cold ammonium hydroxide (3 mL, 0.01 mol), and cold carbon disulfide (0.6 mL, 0.01 mol) was reacted together in a 250 mL round bottom flask for 4 h in an ice bath. Thereafter, 20 mL of cold phenyltin(IV) derivatives (0.005 mol) was added to the already stirring mixture. This mixture was then stirred for an hour. The resulting slightly yellow precipitates were filtered, rinsed with cold ethanol, and dried *in vacuo.*

**[(C_6_H_5_)(Cl)Sn(L)_2_]**: Yield; 2.13 g, 63%; M. Pt, 220–222 °C; Selected IR, (cm^−1^): 1450 ν(C=N), 1325 ν(C_2_–N), 990 ν(C–S), 2945 ν(–C–H), 3350 ν(O–H), 1640 δ(O–H), 454 (Sn–S); ^1^H NMR (600 MHz, CDCl_3_) δ = 7. 80 (S, 2H, -OH), 7.30–7.19 (m, 10H, C_6_H_5_-Sn), 3.96 (t, *J* = 7.1 Hz, 4H, N-CH_2_), 3.86 (t, *J* = 5.2 Hz, 4H, OH-CH_2_), 3.36 (S, 6H, N-CH_3_); ^13^C NMR (151 MHz, CDCl_3_) δ (ppm) = 200.53(-NCS_2_), 150.70, 134.32, 128.3 (C_6_H_5_-Sn-Cl), 60.46 (-CH_2_-OH), 59.90 (-CH_2_-N), 45.13(CH_3_-N); ^119^Sn NMR (CDCl_3_) δ (ppm): −467.4.

C_14_H_21_ClN_2_O_2_S_4_Sn (531,92): C, 31.62; H, 3.98; N, 5.27; O, 6.02; S, 24.12; (found) C, 31.65; H, 4.01; N, 5.29; O, 6.04; S, 24.14.

**[(C6H5)2Sn(L)2]**: Yield; 2.53 g, 70%; M. Pt, 200–202 °C; Selected IR, (cm^−1^): 1424 ν(C=N), 1351 ν(C_2_–N), 985 ν(C–S), 2929 ν(–C–H), 3203 ν(O–H), 1620 δ(O–H), 444 (Sn–S); ^1^H NMR (600 MHz, CDCl_3_) δ = 7. 90 (S, 2H, -OH), 7.41–7.33 (m, 10H, C_6_H_5_-Sn), 3.96 (t, *J* = 4.4 Hz, 4H, N-CH_2_), 3.93 (t, J = 4.3 Hz, 4H, OH-CH_2_), 3.45 (s, *J* = 34.9 Hz, 6H, N-CH_3_); ^13^C NMR (151 MHz, CDCl_3_) δ (ppm) = 200.67(-NCS_2_), 150.80, 134.45, 128.91–128.24(C_6_H_5_-Sn), 60.40 (-CH_2_-OH), 59.98 (-CH_2_-N), 45.20(CH_3_-N); ^119^Sn NMR (CDCl3) δ (ppm): −467.8.

C_20_H_26_N_2_O_2_S_4_Sn (573.99 gmol^−1^): C, 41.89; H, 4.57; N, 4.89; O, 5.58; S, 22.37; Found: C, 41.80; H, 4.51; N, 4.83; O, 5.49; S, 22.29.

### 3.2. Computational Details

The molecular structures of the two Sn complexes were modeled with the GaussView 5.0 and were used as the starting geometries for full geometry optimization using the density functional theory (DFT) model. Based on the free rotation of the ethanoyl and methyl groups on the sp^3^ N on the dithiocarbamate ligand, two possible tagged conformers, syn-syn and anti-anti, were considered, and the geometry optimization was conducted without symmetry constraints. The M06 hybrid functional of Truhlar and Zhao [45] together with the Dunning’s correlation consistent (triple-zeta) basis set (cc-pVTZ) for non-metallic atoms [46,47,48] and LANL2DZ for Sn atom were used. The adopted theoretical model can be coded M06/cc-pVTZ//LANL2DZ. This model has been successfully used for similar systems in a previous study [19]. The molecular structures were fully optimized in the ground state and the optimized geometries were characterized as the true ground state minimum by the absence of a negative vibrational frequency in the force constant calculations. All the calculations were carried out in gas phase at 298 K with the aid of Gaussian 16 [49]. Natural bond orbitals (NBO) calculations were also conducted on the optimized geometry by using the link 607 NBO 7.0 code [50] implemented in Gaussian 16. The atomic dipole moment of the Sn central atom was calculated using the Hirshfeld atomic charge (ADCH) analysis proposed by Lu and Chen [51] and implemented in Multiwfn (freeware). The ADCH calculation was carried out for the possible correlation of the biological activity of the complexes with lipophilicity around the active central metal.

### 3.3. Cytotoxicity Study

An already established procedure was used for the cytotoxicity assay using 3-(4,5-dimethylthiazol-2-yl)-2,5-diphenyltetrazolium bromide (MTT; Sigma), and the cytotoxicity potentials of the as-prepared complexes were evaluated in the following cell lines: immortalized colorectal adenocarcinoma (Caco-2) and human non-tumorigenic immortalized fibroblast (KMST-6). They were acquired from American Type Cell Culture (Manassas, VA, USA). These cells were seeded at a density of 1 × 10^5^ cells/mL in Dulbecco’s modified Eagle medium (DMEM) on a 96 well plate. To supplement the media, 1% penicillin-streptomycin and 10% fetal bovine serum (FBS) were used. These cells were then treated with different concentrations of the complexes (0–100 µM) for 48 h, followed by the addition of the MTT dye (which contained 10 µL of 5 mg/mL per 100 µL of media) for 3 h. Subsequently, 100 µL of DMSO was added to the media to dissolve the insoluble formazan. Camptothecin and 1% DMSO were both used as positive and vehicular control, respectively. The absorbance of each of the wells in 96-well plates was determined at 570 and 700 nm using the POLARstar Omega microplate reader (BMG Labtech, Offenburg, Germany). This study was carried out in triplicate and the percentage of the viability of these cell lines was measured using the formula in Equation (1). Furthermore, changes in the cell morphology because of the complexes added to the assay were monitored and captured using EVOS XL Core Imaging System (Invitrogen, Waltham, MA, USA).

(1) $$\%\;\mathrm{cell}\;\mathrm{viability}=\frac{\mathrm{mean}\;\mathrm{value}\;\mathrm{of}\;\mathrm{test}\;\mathrm{compounds}}{\mathrm{mean}\;\mathrm{value}\;\mathrm{of}\;\mathrm{untreated}}\;\times \;100\;$$

### 3.4. In Vitro Anti-Inflammatory Assay 

Using the same procedure reported in the literature [52], 2 mL of the complexes and the standard drug (Diclofenac) were mixed with 0.2 mL of egg albumin and 2.8 mL of phosphate-buffered saline (pH 6.4). These mixtures were incubated for 20 min at 37 °C and then heated up to 70 °C in a water bath for 5 min. Thereafter, the obtained mixtures were cooled and dispensed into a 96-well plate. The absorbance was then measured at 655 nm using a microplate reader (model 680-Bio-Rad, made in the USA). The used concentration for all the samples and the standard drugs used were 50, 25, 12.5, 6.25, 3.12, and 1.56 µM. All concentrations were carried out in triplicate. The percentage inhibition of the protein denaturation was estimated in terms of percentage inhibition using Equation (2).

(2) $$\%\;\mathrm{Inhibition}=[\frac{\mathrm{absorbance}\;\mathrm{of}\;\mathrm{the}\;\mathrm{test}\;\mathrm{sample}}{\mathrm{absorbance}\;\mathrm{of}\;\mathrm{the}\;\mathrm{control}}-1]\;\times \;100$$

The compounds’ concentration for 50% inhibition (IC_50_) was determined by the dose–response curve.

## 4. Conclusions

Diphenyltin(IV) *N*-methyl-*N*-hydroxyethyldithiocarbamate **[(C6H5)2Sn(L)2]** and chlorophenyltin(IV) *N*-methyl-*N*-hydroxyethyldithiocarbamate **[(C_6_H_5_)(Cl)Sn(L)_2_]** were reported, and spectroscopic and computational studies were used to confirm the mode of coordination. The complexes possess a distorted octahedral geometry around the tin center. The cytotoxicity studies, carried out using some human cell lines, indicated that the complex **[(C6H5)2Sn(L)2]** showed a useful activity and a far better activity than **[(C_6_H_5_)(Cl)Sn(L)_2_]**. Similarly, the anti-inflammatory study revealed the same trend. The observed trends and high activity found for the diphenyltin(IV) derivative **[(C6H5)2Sn(L)2]**, were due to the reduced polarity, which in turn favors permeation through the cell wall, due to the number of phenyl substituents on the tin metal. This was supported by the comparative values of the atomic dipole moment of Sn in the complexes. The cytotoxicity and the anti-inflammatory studies revealed that the diphenyltin complex had a better cytotoxicity and anti-inflammatory properties than the mono-phenyl derivative and both camptothecin and diclofenac, respectively. Thus, the study suggests that **[(C6H5)2Sn(L)2]** could be a promising anticancer agent.

## Figures and Tables

**Table 1:** Selected second-order perturbation energy values.

	[(C_6_H_5_)(Cl)Sn(L)_2_]		[(C6H5)2Sn(L)2]	
	E^(2)^ (kJ/mol)		E^(2)^ (kJ/mol)	
Donor → Acceptor Interactions	Syn-Syn	Anti-Anti	Syn-Syn	Anti-Anti
BD(1) S2-C6 → LP*Sn(1)	42.43	41.97	41.09	40.67
BD(1) S3-C6 → LP*Sn(1)	40.92	40.96	25.94	25.52
BD(1) S4-C20 → LP*Sn(1)	40.58	41.59	23.35	23.68
BD(1) S5-C20 → LP*Sn(1)	42.13	41.55	39.96	39.96
LP(3)S2 → LP*(2)Sn1	202.97	256.10	586.85	285.77

**Table 2:** Atomic orbital compositions of the HOMO, LUMO, HOMO−1, and LUMO+1 of **[(C_6_H_5_)(Cl)Sn(L)_2_]** and **[(C6H5)2Sn(L)2]** (only contributions greater than or equal to 1.0 are recorded).

Composition (%)								
Atom	[(C_6_H_5_)(Cl)Sn(L)_2_]				[(C6H5)2Sn(L)2]			
	HOMO	LUMO	HOMO−1	LUMO+1	HOMO	LUMO	HOMO−1	LUMO+1
Sn1	-	39.26	-	3.53	-	3.10	-	1.51
S2	10.96	7.43	8.80	5.80	10.82	6.81	4.91	5.31
S3	27.07	7.27	19.70	5.50	18.23	11.21	50.60	7.59
S4	8.36	7.48	8.48	10.54	44.10	9.28	28.66	11.56
S5	20.89	7.31	24.37	9.98	14.01	4.55	2.25	7.00
C6	-	1.11	-	15.05	-	24.40	1.13	17.38
N7	1.24	-	-	4.76	-	7.44	-	5.05
C20	-	-	-	27.58	-	18.46	-	23.23
N21	1.08	-	-	8.43	-	5.60	-	6.51
Cl34	17.12	11.90	-	-	-	-	-	-
C34	-	-	-	-	1.13	-	2.62	-
C35	3.79	7.53	2.11	-	1.12	-	-	-
C36	1.20	1.09	6.15	-	-	-	-	-
C37	-	1.09	8.03	-	-	-	-	-
C38	-	-	9.41	-	-	-	-	-
C40	1.12	-	7.76	-	-	-	-	-
C42	2.77	-	-	-	-	-	-	2.00
C45	-	-	-	-	1.21	-	2.70	-
C46	-	-	-	-	1.09	-	-	-
C52	-	-	-	-	1.40	-	-	-

**Table 3:** **The IC_50_** of the mono- and diphenyltin(IV) *N*-methyl-*N*-hydroxyethyldithiocarbamate complexes and camptothecin.

Samples	KMST-6 (µM)	Caco-2 (µM)	PC-3 (µM)
**[(C6H5)2Sn(L)2]**	>100	>100	>100
**[(C6H5)2Sn(L)2]**	11.81 ± 0.15	4.937 ± 0.12	1.630 ± 0.10
Camptothecin	59.91 ± 0.21	>100	24.41 ± 0.11

## Data Availability

Data available upon request from corresponding authors.

## Supplementary Materials

The following supporting information can be downloaded at: https://www.mdpi.com/article/10.3390/molecules27092947/s1, Figure S1: 1H and 13C spectra of (a) **[(C6H5)2Sn(L)2]** and (b) **[(C6H5)(Cl)Sn(L)2**; Table S1: Gaussian input file for conformer A of **[(C6H5)2Sn(L)2]**; Gaussian input file for conformer B of **[(C6H5)2Sn(L)2]**; Gaussian input file for conformer A of **[(C6H5)2Sn(L)2]**; Gaussian input file for conformer B of **[(C6H5)2Sn(L)2]**.

## Sample Availability

Samples of the compounds might be available upon agreement on certain terms and conditions from the authors.
