# Supplementary material for: Synthesis, Theoretical Calculation, and Biological Studies of Mono- and Diphenyltin(IV) Complexes of N-Methyl-N-hydroxyethyldithiocarbamate
Source: Molecules. 2022 May 5;27(9):2947. doi: 10.3390/molecules27092947 (PMC9105561; doi:10.3390/molecules27092947)
Supplement: Supplementary file 1 [file molecules-27-02947-s001.zip › molecules-1687133-supplementary.pdf]

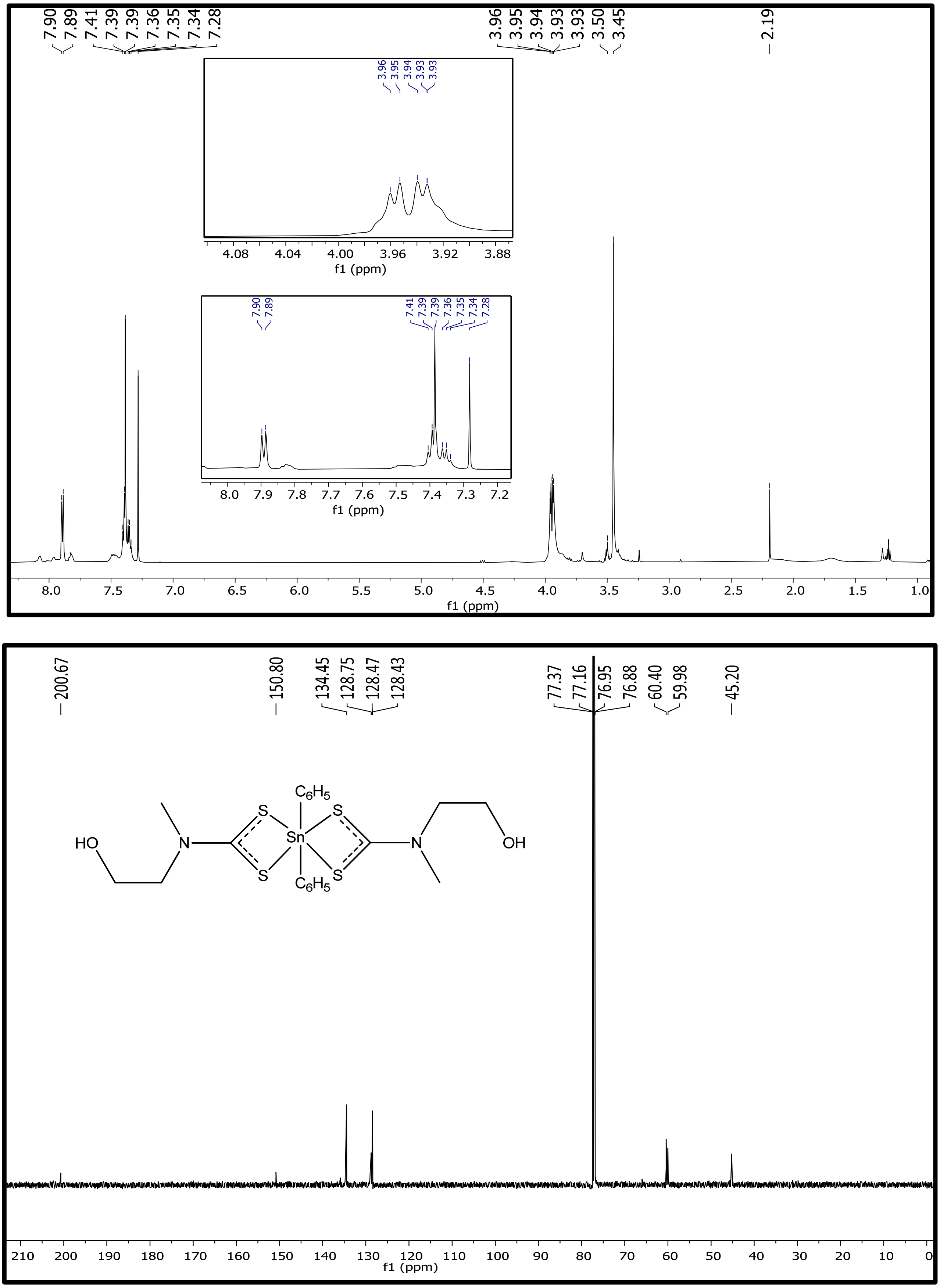
7.90 7.89 7.41 7.39 7.39 7.36 7.35 7.34 7.28
3.96 3.95 3.94 3.93 3.93 3.50 3.45
2.19
4.08 4.04 4.00 3.96 3.92 3.88
f1 (ppm)
8.0 7.9 7.8 7.7 7.6 7.5 7.4 7.3 7.2
f1 (ppm)
8.0 7.5 7.0 6.5 6.0 5.5 5.0 4.5 4.0 3.5 3.0 2.5 2.0 1.5 1.0
f1 (ppm)
200.67
150.80
134.45 128.75 128.47 128.43
77.37 77.16 76.95 76.88 60.40 59.98
45.20
HO
N
S
Sn
C_6H_5
OH
210 200 190 180 170 160 150 140 130 120 110 100 90 80 70 60 50 40 30 20 10 0
f1 (ppm)

(a)

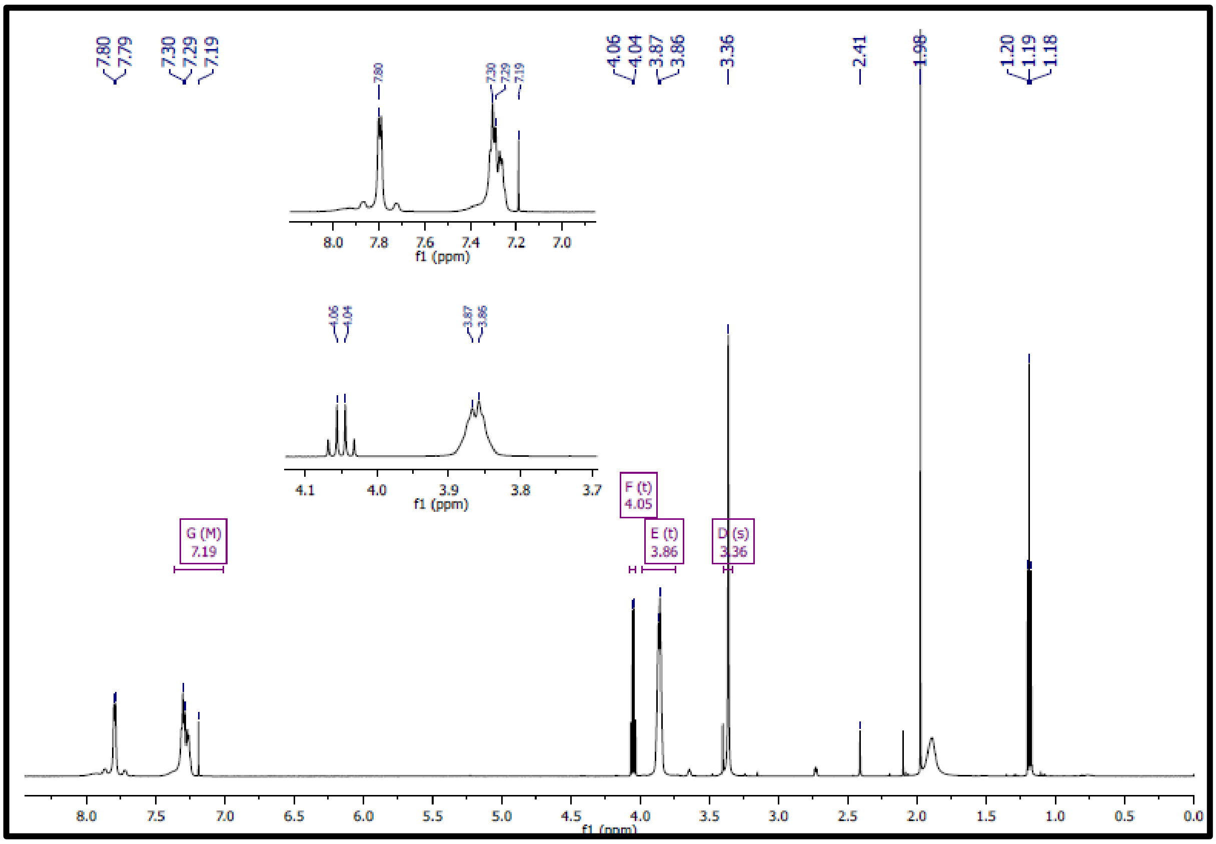

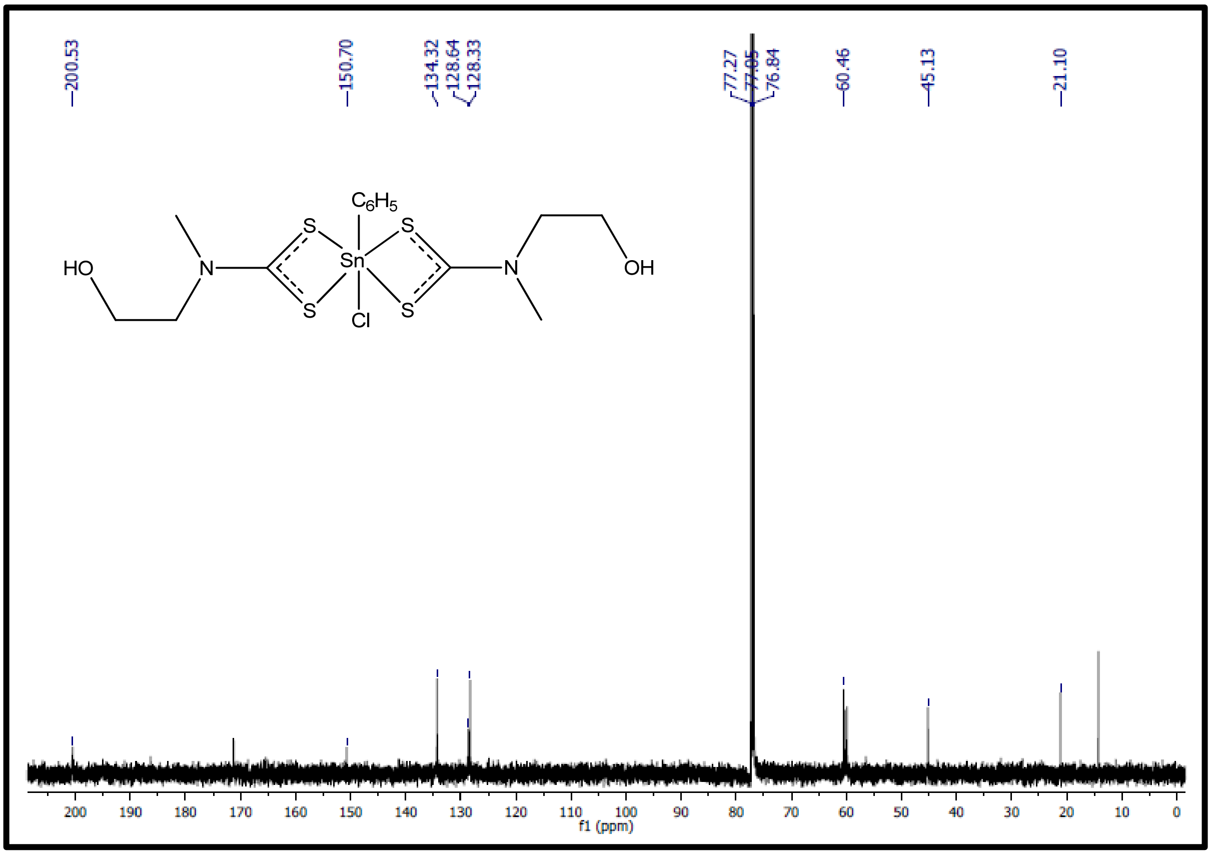

(b)

Figure S1. ^{1}H and ^{13}C spectra of **(a) [$(C_6H_5)_2Sn(L)_2$]** and **(b) [$(C_6H_5)(Cl)Sn(L)_2$**

Table S1. Gaussian input file for conformer A of $[(C_6H_5)_2Sn(L)_2]$

```
%mem=10GB
%CPU=0-23
%chk=Sncomplx_ph_cis.chk
# opt freq genecp m06

Sncomplx_ph_cis

0 1
 Sn     0.03602369    0.23682095   -0.19792621
 S     -1.32976711   -0.29117480    1.86099494
 S     -2.86002604    0.36528424   -0.62733089
 S      2.67062277    0.54379565   -1.54091413
 S      2.03095240   -0.18694520    1.29502620
 C     -2.84624659   -0.04211809    1.01364285
 N     -3.96377195   -0.19478392    1.73580510
 C     -3.91448186   -0.47248789    3.16408186
 H     -2.99767444   -0.04096803    3.57051031
 H     -4.76117810    0.00550277    3.65403128
 C     -3.93711956   -1.95890527    3.43640383
 H     -3.14290744   -2.45092986    2.85479924
 H     -4.89767358   -2.36601851    3.09893800
 O     -3.74356678   -2.12004747    4.81799216
 H     -3.95055388   -3.02217688    5.06233305
 C     -5.28091654   -0.01711719    1.14542309
 H     -5.45871101   -0.74989411    0.35660063
 H     -6.03038016   -0.14560544    1.92254137
 H     -5.38924760    0.97901051    0.71357574
 C      3.18801093    0.12659156    0.00799738
 N      4.49130652    0.01805816    0.30633139
 C      4.98199761   -0.38510785    1.61531213
 H      5.92129148    0.14407102    1.80332274
 H      4.27751354   -0.07792846    2.38718545
 C      5.20513975   -1.88437654    1.69300255
 H      4.24811381   -2.39449898    1.56444395
 H      5.85597212   -2.20714365    0.86824973
 O      5.71048473   -2.26606038    2.94439343
 H      6.61013061   -1.94487824    3.02787156
 C      5.48401983    0.20478813   -0.73892074
 H      6.47417846    0.07621316   -0.30663798
 H      5.34436408   -0.51727531   -1.54509616
 H      5.40239731    1.20227560   -1.17227069
 C     -0.10323978   -1.48871374   -1.42053492
 C     -0.17652414   -1.38909859   -2.80425394
 C     -0.14820146   -2.74156421   -0.82671957
 C     -0.29524982   -2.52596060   -3.58344983
 H     -0.13471790   -0.41715966   -3.28722649
 C     -0.26099496   -3.88222849   -1.60753504
 H     -0.09552164   -2.83827706    0.25301062
 C     -0.33631425   -3.77504870   -2.98445828
 H     -0.35259392   -2.43838022   -4.66113320
 H     -0.29011927   -4.85674484   -1.13618317
 H     -0.42554933   -4.66528704   -3.59398339
 C     -0.03281228    2.34079236   -0.44074079
 C     -0.52918907    2.91803930   -1.60251940
 C      0.45533436    3.15798458    0.56873156
```

```
C         -0.53180839    4.29324388   -1.75591152
H         -0.92800564    2.29597427   -2.39800060
C          0.44179995    4.53638504    0.42049570
H          0.85129612    2.72374149    1.48131844
C         -0.04804670    5.10403828   -0.74208937
H         -0.91752477    4.73387592   -2.66661108
H          0.81709838    5.16724289    1.21658917
H         -0.05546997    6.18025067   -0.85901215

C H N O S 0
cc-pVTZ
****
Sn 0
LANL2DZ
****

Sn 0
LANL2DZ
```

Gaussian input file for conformer B of $[(C_6H_5)_2Sn(L)_2]$

```
%mem=10GB
%CPU=0-23
%chk=Sncomplx_ph_new.chk
# opt freq genecp m06

Sncomplx_ph_cis

0 1
Sn         0.00350940   -0.00534824   -0.22932078
S          1.78299587   -1.83929104   -0.16929713
S          2.30109057    1.11251539   -0.25298463
S         -1.78346176    1.82635658   -0.26885192
S         -2.29344910   -1.12618958   -0.17351114
C          2.88654872   -0.51305742   -0.20618026
N          4.19424558   -0.76013419   -0.19280287
C          5.18522462    0.30746628   -0.16617447
H          6.07363902   -0.03030463   -0.70209169
H          4.79763601    1.18093013   -0.68914564
C          5.55522233    0.67438128    1.25342093
H          5.87131238   -0.23004197    1.79412639
H          4.66857754    1.07076058    1.76328129
O          6.58878254    1.61762560    1.16369577
H          6.74270994    2.00311513    2.02662455
C          4.68578499   -2.12654798   -0.09378012
H          4.34030318   -2.72050074   -0.94097219
H          5.77241556   -2.10655649   -0.09032731
H          4.33369350   -2.60200264    0.82318596
C         -2.88209677    0.49691491   -0.22671151
N         -4.19174613    0.74084686   -0.23103898
C         -5.18069557   -0.32622800   -0.14636589
H         -6.05432797   -0.02292019   -0.72991948
H         -4.78204010   -1.23204083   -0.60182491
C         -5.57755159   -0.60454154    1.29281343
H         -4.70056439   -0.94788641    1.84568634
```

```
 H              -5.91725973   0.32391666   1.77265026
 O              -6.53819187  -1.62116090   1.36703576
 H              -7.37694056  -1.28630754   1.04485693
 C              -4.68569322   2.10932357  -0.20779873
 H              -5.77246821   2.08899026  -0.22189612
 H              -4.35017553   2.63084980   0.69023024
 H              -4.32695322   2.66140051  -1.07746302
 C               0.00780523   0.06482999   1.93953987
 C               0.12626757   1.28934326   2.59757129
 C              -0.10796998  -1.11418602   2.67579085
 C               0.12962354   1.33466955   3.99154260
 H               0.21829896   2.21860195   2.01683874
 C              -0.10562104  -1.06892170   4.07019234
 H              -0.20152443  -2.07936223   2.15732054
 C               0.01328600   0.15521462   4.72813489
 H               0.22362415   2.29974512   4.51028100
 H              -0.19733353  -1.99864539   4.65043100
 H               0.01589750   0.19120717   5.82722272
 C              -0.00459955  -0.06776420  -2.39840780
 C               0.16565503   1.10750815  -3.13067804
 C              -0.17990371  -1.28280879  -3.06058846
 C               0.15991813   1.06774839  -4.52481146
 H               0.30306885   2.06537649  -2.60832999
 C              -0.18468144  -1.32289462  -4.45514222
 H              -0.31392653  -2.20922785  -2.48360090
 C              -0.01494766  -0.14787632  -5.18730302
 H               0.29348113   1.99415993  -5.10206278
 H              -0.32251099  -2.28110778  -4.97697053
 H              -0.01937679  -0.17898660  -6.28653406

C H N O S 0
cc-pVTZ
****
Sn 0
LANL2DZ
****

Sn 0
LANL2DZ
```

Gaussian input file for conformer A of [(C_6H_5)(Cl)Sn(L)$_2$]

```
%mem=10GB
%CPU=0-23
%chk=Sncomplx_Clph_cis.chk
# opt freq genecp m06

Sncomplx_Clph_cis

0 1
 Sn             -0.00531159  -0.18538193  -0.00061126
 S              -1.82576199  -0.23254840   1.85094375
 S              -2.35236716  -0.23600488  -1.11102484
 S               1.82392790  -0.24916921  -1.84927723
 S               2.34044025  -0.25602360   1.11425515
```

C	-2.92423916	-0.21807786	0.51979785
N	-4.23498356	-0.20839437	0.77100216
C	-4.76782031	-0.08556753	2.12079579
H	-4.05835477	0.48427488	2.72329335
H	-5.70341914	0.47128272	2.09726627
C	-4.99062895	-1.44318515	2.74803334
H	-4.06062042	-2.02830579	2.69385092
H	-5.75386312	-1.98152124	2.17319043
O	-5.38402756	-1.21083699	4.07475229
H	-5.69970285	-2.02908675	4.45894457
C	-5.19675700	-0.21278099	-0.32052262
H	-5.09821809	-1.11977306	-0.91860657
H	-6.19871376	-0.16748308	0.09870670
H	-5.04469737	0.64468438	-0.97803882
C	2.91634552	-0.23426672	-0.51408736
N	4.22925271	-0.22101644	-0.76093639
C	5.22248094	-0.25693920	0.30407369
H	6.08955154	0.32357853	-0.02406236
H	4.82279357	0.22566944	1.19490940
C	5.63680346	-1.68014568	0.63267148
H	4.76983094	-2.22706400	1.00884509
H	5.96947259	-2.19246424	-0.28094796
O	6.61225769	-1.70746562	1.63883384
H	7.43967458	-1.37972927	1.28197519
C	4.72334827	-0.28255284	-2.12743819
H	5.81045007	-0.28532822	-2.10554837
H	4.37418696	-1.18638250	-2.62941317
H	4.37890880	0.57832100	-2.70215712
Cl	-0.01462071	-2.59730485	-0.00597580
C	0.00727251	1.97664287	-0.00466490
C	-0.19911511	2.68072334	-1.18173122
C	0.21998890	2.68199725	1.17051307
C	-0.19320447	4.06659242	-1.18686196
H	-0.36886104	2.15226499	-2.11530767
C	0.22681394	4.06790566	1.17179447
H	0.38416143	2.15457398	2.10565892
C	0.02002569	4.76122048	-0.00847959
H	-0.35624991	4.60411013	-2.11283117
H	0.39399601	4.60641917	2.09644617
H	0.02464514	5.84391937	-0.00990617

C H N O Cl S 0
cc-pVTZ
****
Sn 0
LANL2DZ
****

Sn 0
LANL2DZ

Gaussian input file for conformer B of [(C_6H_5)(Cl)Sn(L)$_2$]

%mem=10GB

```
%CPU=0-23
%chk=Sncomplx_Cl.chk
# opt freq genecp m06

Sncomplx_Cl

0 1
 Sn      -1.31715670   1.37373368  -0.54579608
 S       -1.32111749   1.37375680  -2.95599589
 S       -3.72715217   1.37373368  -0.54112295
 S       -1.11821625   1.38524547   1.82718094
 S        1.09121442   1.37589272  -0.51920488
 C       -3.19640868   1.31288680  -2.41806065
 N       -4.17172175   1.29887522  -3.51781884
 C       -5.08899245   2.43851560  -3.37382483
 H       -5.68114595   2.31052432  -2.49185405
 H       -4.52373986   3.34365693  -3.29566667
 C       -6.01122503   2.51390910  -4.60484184
 H       -6.59666164   1.62044394  -4.66716342
 H       -5.41773365   2.61387598  -5.48953083
 O       -6.87834824   3.64438700  -4.48229768
 H       -7.51581946   3.63858658  -5.20006979
 C       -4.93793093   0.04500858  -3.47729800
 H       -5.61277239   0.06606826  -2.64721035
 H       -5.49305181  -0.06326934  -4.38560230
 H       -4.26568522  -0.78045126  -3.36958659
 C        0.79591324   1.49867199   1.46243553
 N        1.95206931   1.57322994   2.36722516
 C        2.06044555   0.32014115   3.12809510
 H        2.90588085   0.37184460   3.78190387
 H        1.17132948   0.17545855   3.70553735
 C        2.23685019  -0.85879756   2.15311566
 H        1.28839802  -1.32240444   1.97873922
 H        2.63378007  -0.49924079   1.22679736
 O        3.13773567  -1.81446833   2.71879998
 H        4.01972622  -1.43772627   2.76072963
 C        1.77458985   2.69753552   3.29746061
 H        2.62966840   2.77004225   3.93658771
 H        1.66605718   3.60590498   2.74249613
 H        0.89885826   2.53503105   3.89040790
 Cl      -1.39518704   3.76242511  -0.55862245
 Cl      -1.39205614  -1.01509197  -0.54723678

C H N O Cl 0
cc-pVTZ
****
Sn 0
LANL2DZ
****

Sn 0
LANL2DZ
```
